# Bridging the know-do gap: improved adolescent sexual and reproductive health and health information systems in West Africa and the Middle East

**DOI:** 10.1186/s12978-025-01987-2

**Published:** 2025-05-31

**Authors:** Chaitali Sinha, Marie-Gloriose Ingabire, Anne-Marie Schryer-Roy

**Affiliations:** 1 International Development Research Centre, Ottawa, Canada; 2 International Development Research Centre, Dakar, Senegal; 3 International Rescue Committee, Nairobi, Kenya

**Keywords:** Sexual and reproductive health, Health information systems, Implementation research, Adolescent health

## Abstract

**Background:**

As the world approaches the final five years to achieve the Sustainable Development Goals (SDGs), a global polycrisis threatens progress on health and well-being. Populations experiencing vulnerabilities, particularly women, girls, and adolescents in low- and middle-income countries, shoulder a disproportionate burden. Universal access to sexual and reproductive health (SRH) services has seen mixed progress. In particular, adolescents are facing heightened risks such as early marriage, unintended pregnancies, and limited access to SRH services, compounded by gender norms and fragile contexts. Integrated, community-driven, and gender-transformative approaches are urgently needed, as are robust health data and information systems. However, fragmented systems suffer from poor data quality and underrepresentation of marginalized groups. Digital innovations hold promise for addressing these gaps but must be adapted to local contexts.

**Bridging the Know-Do Gap:**

This supplement presents findings from the Cedar Cohort, an initiative supported by the International Development Research Centre across West Africa and the Middle East (2017–2023). The cohort's 16 implementation research projects explored SRH services for adolescents and health information systems, emphasizing gender equality, human rights, and knowledge translation.

The articles are organized around three themes: (1) adopting contextual approaches rooted in local communities and their context-specific needs, (2) advancing gender equality and inclusion and (3) harnessing the transformational potential of data and information systems. Authored by LMIC-based researchers, the supplement provides concrete findings and localized insights into the transformative change possible when high-quality research is combined with an understanding of social, economic, political, and cultural factors at the individual, organizational, structural and systems levels.

**Conclusion:**

The collection of research projects featured in this supplement enriches our understanding of designing and implementing effective initiatives to improve health outcomes for women, children and adolescents. The findings emphasize that no singular solution exists for the multifaceted SRH challenges that continue to evolve. The projects in the Cedar Cohort offer critical insights into designing and scaling SRH interventions that are locally relevant, inclusive, and impactful. The current polycrisis necessitates a re-evaluation of global strategies, and the findings from the research projects in this supplement provide indications of ways forward.

## Résumé

**Contexte:**

Alors que le monde approche des cinq dernières années pour atteindre les objectifs de développement durable (ODD), une polycrise mondiale menace les progrès en matière de santé et de bien-être. Les populations vulnérables, en particulier les femmes, les filles et les adolescentes des pays à revenu faible et intermédiaire, supportent une charge disproportionnée. L’accès universel aux services de santé sexuelle et reproductive (SSR) a connu des progrès mitigés. En particulier, les adolescentes font face à des risques accrus tels que le mariage précoce, les grossesses non désirées et l’accès limité aux services de SSR, aggravés par les normes sexospécifiques et les contextes fragiles. Il est urgent d’adopter des approches intégrées, communautaires et transformatives sensible au genre , tout comme de solides systèmes d’information et de données sur la santé. Cependant, les systèmes fragmentés souffrent d’une mauvaise qualité des données et de la sous-représentation des groupes marginalisés. Les innovations numériques sont prometteuses pour combler ces lacunes, mais elles doivent être adaptées aux contextes locaux.

**Combler l’écart « savoir-action »:**

Ce supplément présente les résultats de la cohorte Cedar, une initiative soutenue par le Centre de recherches pour le développement international en Afrique de l’Ouest et au Moyen-Orient (2017-2023). Les 16 projets de recherche de mise en œuvre de la cohorte ont exploré les services de SSR pour les adolescents et les systèmes d’information sanitaire, en mettant l’accent sur l’égalité des sexes, les droits de l’homme et le transfert des connaissances. Les articles s’articulent autour de trois thèmes, à savoir l’adoption d’approches contextuelles ancrées dans les communautés locales et leurs besoins propres au contexte, la promotion de l’égalité entre les sexes et de l’inclusion, et l’exploitation du potentiel de transformation des données et des systèmes d’information. Rédigé par des chercheurs basés dans les pays à revenu faible ou intermédiaire, le supplément fournit des résultats concrets et des aperçus localisés sur le changement transformateur possible lorsque des recherches de haute qualité sont combinées à une compréhension des facteurs sociaux, économiques, politiques et culturels aux niveaux individuel, organisationnel, structurel et systémique.

**Conclusion:**

La collection de projets de recherche présentée dans ce supplément enrichit notre compréhension de la conception et de la mise en œuvre d’initiatives efficaces visant à améliorer la santé des femmes, des enfants et des adolescents. Les résultats soulignent qu’il n’existe pas de solution unique aux défis multiformes de la SSR qui continuent d’évoluer. Les projets de la cohorte Cedar offrent des perspectives essentielles pour la conception et la mise à l’échelle d’interventions pertinentes, inclusives et efficaces au niveau local en matière de SSR. La polycrise actuelle nécessite une réévaluation des stratégies mondiales, et les résultats des projets de recherche présentés dans ce supplément fournissent des indications sur les voies à suivre.

## Background

As the world approaches the last five years to achieve the Sustainable Development Goals (SDGs), we collectively find ourselves facing a polycrisis that is cause for deep concern for global health targets and improved health and well-being for all [1]. Contributing phenomena to the polycrisis include increased frequency and intensity of extreme climate events, proliferating and prolonged conflicts, forced displacement, migration, rollback of women’s rights and efforts to enhance gender equality and inclusion, economic turmoil, and a growing ‘infodemic’, which can seed mistrust in health authorities and undermine public health responses [2]. These concomitant phenomena are contributing to significant declines in key social determinants of health and to increased morbidity and mortality among populations experiencing vulnerabilities—including women and girls [3, 4]. The impacts range from disruptions in accessing safe and affordable sexual and reproductive health (SRH) services, increased vulnerabilities and inequalities, and challenges to overall well-being. There is an urgent need for integrated, locally championed and gender-transformative strategies that address sexual and reproductive health within the broader context of global health challenges.

### Addressing unmet adolescent sexual and reproductive health (SRH) needs

Universal access to SRH services, captured in Target 3.7 of the SDGs, has experienced mixed progress to date—largely due to setbacks for women based in low- and middle-income countries (LMICs), including those in humanitarian settings [1, 5, 6]. Improving SRH is a shared responsibility across households, communities and societies, including women, men, girls and boys [7]. However, evidence has shown SRH gets deprioritized, even by women themselves, during global crises such as the COVID-19 pandemic and in conflict or humanitarian settings, relative to other health rights and needs [8, 9]. Something else, or someone else, seems to be always more important or less able to wait. The SRH needs of women and adolescents are never quite urgent enough for the world. This carries calamitous repercussions for women and girls when attempting to access menstrual hygiene products, modern contraceptives, prenatal care and safe delivery and postnatal care.

Adolescents (aged 10–19 years) represent a large and growing demographic at 1.3 billion across the globe, among which 90% reside in LMICs [10]. With the largest cohort of youth in history entering their reproductive age, many are deprived of essential information and access to SRH services needed to exercise their rights and actualize their potential in their adolescence and throughout their life course [11, 12]. Adolescent girls face high risks of early marriage, unwanted pregnancies, sexual and gender-based violence and transactional sex [13, 14]. Gender inequalities, and negative social norms and practices can compound those risks, which are further exacerbated in fragile and conflict settings [12, 15]. Targeted and tailored policies and interventions are needed to address unmet SRH needs among adolescent girls and boys in LMICs—particularly those experiencing acute or prolonged deprivation and upheaval in their lives.

### Strengthening health data and health information systems

Effectively addressing these gaps in SRH services requires valid, timely and reliable data and information systems [16]. Yet, health data and health information systems used to improve SRH are plagued by poor coverage, incomplete records, inferior data quality, untimely reporting, limited analysis and use, and fragmented systems [17, 18]. Efforts to strengthen health information systems (HIS) often, and sometimes even intentionally, fail to capture marginalised groups, including adolescents, which are subsequently underrepresented for planning and resource allocation purposes [19, 20]. Although digital health innovations, including widespread use of mobile phones, targeted localised applications, big data, and the advent of artificial intelligence solutions can play a pivotal role in pre-empting or overcoming some of these challenges, there are context-specific aspects related to health seeking behaviour, health provider interactions, and protecting privacy and respectful care that require continued attention [21, 22].Partial and biased capture of health data leads to partial and biased approaches to allocating resources, planning health services and measuring progress related to Target 3.7, and other related targets, within the SDGs.

### Real-world application of programs and policy recommendations

There is no shortage of global, regional and national strategy and policy documents are focused on adolescent health and well-being, and on strengthening health data and information systems. However, there are significant gaps between what is stated in these documents and what happens when the policy prescriptions and program frameworks are rolled out in the real world [23, 24]. These gaps, often referred to as the ‘know-do’ gap, can be filled by well-designed and delivered implementation research efforts that seek to understand how and why (and which, when, where and for whom) interventions ‘work’ in the real world – and to test approaches to improve them [25].

For example, comprehensive sexuality education (CSE) has been demonstrated as an effective means of providing accurate, sensitive and age-appropriate information on SRH to improve adolescents’ decision making and health behaviours [26, 27]. Different forms of information and education can be provided through CSE programs to support adolescents in navigating menstrual health, pregnancy prevention, contraception, sexually transmitted infections, as well as strengthening decision-making, consent, negotiation and other life skills. Although the success of CSE programs has been documented through improvements in SRH knowledge and reductions in risky practices that result in pregnancy and sexually transmitted disease, implementing these programs in real-world settings across different cultural contexts have surfaced many challenges related to adaptation and subsequent adoption [28].

The use of implementation research is not limited to the introduction of tangible interventions. It can be applied to critical, yet less tangible, elements such as policies and processes. Implementation outcomes for health research are different from health outcomes. The former measure the success (or failure) of an intervention (for example, its acceptability, adoption, appropriateness, feasibility, fidelity, cost, coverage and sustainability), whereas health outcomes measure changes in health status (for example, mortality, morbidity, quality of life, Disability-Adjusted Life Years (DALYs), satisfaction with care or effectiveness of care). Therefore, using implementation research as a lens to generate locally relevant evidence on how different adolescent SRH programs and efforts strengthen SRH information systems, can help fill some of the ‘know-do gaps’. Specifically, adopting approaches that are community-based, cognizant and responsive to social and gender norms, and aim to strengthen—rather than fragment—health systems, can help reduce health inequities and support greater gender equality and inclusion.

### Cedar Cohort: bridging the know-do gap in West Africa and the Middle East

Across West Africa and the Middle East, countries are grappling with a combination of emerging and protracted conflicts, large numbers of internally and externally displaced populations, climate shocks, disease outbreaks, health worker shortages, high rates of out-of-pocket payments, rural and urban divides, and regressive social and gender norms related to SRH [29, 30]. From 2017 to 2023, the International Development Research Centre (IDRC) supported a cohort of 16 implementation research projects in West Africa and the Middle East, across 11 countries. The implementation research approach adopted by each individual project, and by the cohort as a whole, proved critical in responding to contextual shifts and enabling the necessary recalibrations by the locally based research teams.

With a dual focus on adolescent SRH and on health information systems, the Cedar Cohort projects included cross-cutting areas of research on issues of gender equality, human rights, fragile settings, research ethics, and knowledge translation. Each project was rooted in approaches that understand local contexts, champion community mobilization and leadership, and translate knowledge toward achieving positive changes at scale. Program and policy solutions were co-created with under-served, and in many cases excluded, populations, which successfully seeded change processes to address unmet priority needs. All 16 projects are now complete and 10 of the projects are represented in this supplement. As a cohort, these projects provide real-world, concrete findings and localized insights into the transformative change possible when high-quality research is combined with an understanding of social, economic, political, and cultural factors that shape sexual and reproductive health and rights at the individual, organizational, structural and systems’ levels.

### Supplement overview

The research articles featured in this supplement are grouped under three themes: 1) Context: rooted in local priorities, people and partnerships; 2) Gender equality and inclusion: central considerations, not on the periphery; and 3) Transformative potential of data and information systems: representation, reliability and reach.

To begin, a commentary by Wick et al. sets the tone for the supplement by reflecting on the reality of SRH across the Arab world and Turkey, 30 years after the landmark 1994 International Conference on Population and Development (ICPD) in Cairo, Egypt. This piece reflects on what context has meant to the work of the Reproductive Health Working Group, and how it has shaped the work of the regional network in three different ways: as an object of study, as something that structures the network’s activities, and as a way to conceptualize network support.

### Theme 1—context: rooted in local people, priorities and partnerships

The importance of considering context is widely accepted by those designing, implementing and reporting on efforts to improve SRH outcomes. However, the passing reference to ‘context’ or the limited input and leadership from local actors and institutions continues to hamstring the uptake and sustainability of many such initiatives [31]. Careful and ongoing consideration of context is central to implementation research projects. This includes examining cultural, social, economic and geo-political contexts—including local values, norms and practices—within which specific populations are seeking SRH information and services. Papers in this section adopt contextually grounded approaches that are rooted in local communities (people), their context-specific needs (priorities) and different local collaborations (partnerships) to improve health outcomes through measuring a range of implementation outcomes.

In Nigeria, as in many other countries, conventional healthcare structures often operate in silos. The  lack of coordination and collaboration across sectors impedes the effectiveness of health interventions, including SRH. To tackle this challenge, Eze et al. explored the potential for a community-embedded intervention to strengthen intersectoral collaboration to improve adolescent SRH outcomes. The collaborations formed, which helped increase the acceptability and adoption of adolescent SRH strategies, emphasized the significance of trust-building, shared goals, and clear communication. Specifically, the study also identified the pivotal role of local champions and intermediaries—namely community leaders—in facilitating collaboration and in helping to overcome barriers to healthcare access.

In the Gambia, where early marriage is common, Lowe et al. uncovered the social and cultural factors contributing to this situation in rural communities and used that knowledge to design a package of interventions to address early marriage among girls aged 10–19. The authors found that the interventions that had been carefully designed with meaningful participation from communities successfully contributed to both an increase in the mean and average age of marriage, as well as to a change in knowledge and attitudes towards early marriage among parents.

In Ghana, Fenny et al. adopted an established framework, developed by the West African Health Organization (WAHO), and consulted with a wide range of stakeholders to list, rank, validate and prioritize existing interventions aimed at addressing the needs of adolescents in Ghana. In the process, they identified some common denominators between successful interventions, including the presence of activities that address key behavioural and lifestyle changes, as well as efforts to encourage peer-education.

### Theme 2—gender equality and inclusion: central considerations, not on the periphery

Gender inequality is among the most pervasive drivers of inequalities in health, and one that is the subject of much backlash and resistance to change [32]. Marginalized and disadvantaged groups are consistently exposed to health risks, are less likely to seek health care, receive lower quality health services and experience poorer health outcomes [33]. Within these groups, intersecting identities and experiences related to education, income, race, ethnicity and other socioeconomic characteristics reveal compounded risks and vulnerabilities to greater health inequalities [34, 35]. For example, adopting an intersectional lens to explore gender relations and norms can strengthen how SRH programs are designed and delivered for adolescents to promote agency, healthy choices and to equip them for the future [36].

As part of a multi-country project in Burkina Faso, Kenya and Nigeria, Maina et al. explored the use of radio programming to improve young adolescents’ sexual and reproductive health knowledge and practices in Kenya. Gathering data through in-depth interviews with adolescent girls and boys, as well as their parents, the study reveals that open discussions of gender norms affecting adolescent SRH are very limited within the community and within households. In the informal settlement, the airing of the radio program created an opportunity for inequitable gender norms to be debated and challenged, and for relationships between adolescents and their parents to be strengthened.

In Ghana, Klu et al. looked at the gender dynamics that affect opinions on condom use among adolescent girls and boys among adolescents aged 10–19 years old across 30 communities. The study found that gender, age and employment status all had an influence not only on condom use, but also on sexual experiences more broadly. Girls, for example, were found to have less sexual bargaining power in terms of condom usage due to limited access to resources (to purchase condoms), as well as conservative social and cultural norms with regards to premarital sex.

### Theme 3—transformative potential of data and information systems: representation, reliability and reach

Achieving the SDGs by 2030 relies on robust and reliable data and information systems [37]. Health information systems are part of the health system, as well as health-adjacent systems such as statistical systems and education systems—and are collected through both paper-based and electronic systems [38]. Health data are complex and should not be viewed as neutral structured inputs. They tend to reflect and reinforce dominant societal structures and biases, which can exacerbate various intersecting inequalities [39]. Representation in health data is an important step towards greater awareness and responsiveness to the pressing health needs of different population groups seeking to meet their SRH needs.

Across many LMIC contexts, there is a lack of information regarding the health needs of adolescents. In the West Bank, Shalash et al., undertook the first comprehensive mapping of available adolescent health services, paving the way for the development of policies and programs that address the needs of adolescents in a humanitarian crisis and tackle existing gaps in services.

In Jordan, the existence of multiple systems to capture and store primary data on antenatal care services has led to challenges in terms of the quality and continuity of care for women, with information not always being available when needed. In their article, Amiri et al., evaluated the newly created *harmonized* Reproductive Health Registry (hRHR) to help reduce fragmentation and improve continuity of care. Despite some challenges, including lack of connectivity and poor information technology infrastructure, the new system was found to improve data capture, reduce the time involved with recording information, and enhance access to patient data.

The lack of data on stillbirths and neonatal deaths remains a problem across many LMICs. This data scarcity impedes efforts to identify possible causes of death and associated risk factors, information that is critical to the development of interventions to reduce these deaths. In Jordan, Khader et al. reviewed data on all deaths that occurred across five large hospitals in Jordan over a period of one and a half year and identified preventable causes of stillbirths and neonatal deaths that can be addressed with simple interventions.

In Ghana, where under five mortality remains high, Ruhama et al. tested a mobile-based information system to monitor childhood conditions, offer telemedicine consultations, in rural and remote areas, and deliver health promotion messages. Their implementation research project demonstrates how a customized and low-cost intervention, linking community, health provider and health facility information, can help improve child health outcomes and contribute to reducing the burden of prevalent illnesses in a resource-constrained setting.

Recognizing the transformational potential of data to influence decision-making, El-Jardali et al. assessed the national health information system in Jordan with an eye on identifying areas that required improvement when equipping decision-makers with credible and timely data and related analysis. Promoting data-informed decision-making is identified as a priority in Jordan’s National Health Sector Strategy. The authors identified a set of core health systems indicators to be included in the HIS and developed a set of tools and manuals that can be adapted by other countries to strengthen their HIS and promote data-driven health policymaking.

### Contribution to the field

The results from the cohort of research projects featured in this supplement significantly enrich our understanding of designing and implementing effective initiatives aimed at enhancing child, adolescent, and maternal health in LMICs.

The findings emphasize that no singular solution exists for the multifaceted challenges of enhancing maternal, child, and adolescent health. Instead, a multipronged strategy is essential, encompassing multidisciplinary and multisectoral collaboration and socio-economic, political, and legal interventions that are both data-driven and gender-transformative. The journey toward improved health outcomes requires insights on if, how, for whom, and in what contexts certain strategies can contribute to positive change. By adopting an implementation research approach, this collection examines how implementation outcomes – such as acceptability, adoption, feasibility, cost-effectiveness and sustainability – can drive measurable and meaningful solutions.

This supplement highlights crucial prerequisites for improved health outcomes and reduced health inequities success, such as a clear understanding of the root causes, identification of key stakeholders and gatekeepers, recognition of potential change agents, and a grasp of power dynamics. Moreover, it contends that true and transformative change is contingent on meaningful community engagement, crucial for understanding, co-designing, and co-implementing relevant and sustainable approaches that leverage indigenous knowledge and relevant existing partnerships. Additionally, locally generated data, locally designed data systems and locally championed data-informed decision-making processes, which take into account social and gender considerations, are discussed as essential ingredients to improve sexual and reproductive health throughout the life course – from newborns, children, adolescents and women. Lastly, the integration of evidence-based social and technological innovations can greatly facilitate unlocking transformative positive change.

These collective contributions from the research projects provide a robust framework for advancing implementation research in the field of women, child, and adolescent health.

## Conclusion

Evidence and impact generated from this cohort are rooted in approaches that understand local contexts, champion community mobilization and leadership, and translate knowledge toward achieving positive changes at scale. Positioning the voices, experiences and co-created solutions from under-served, and in many cases excluded, populations opened space for dialogue and exchange and seeded change processes to address unmet priority needs.

Authored by LMIC-based researchers, this supplement provides concrete findings and localized insights into the transformative change possible when high-quality research is combined with an understanding of social, economic, political, and cultural factors that shape sexual and reproductive health and rights at the individual, organizational, structural and systems’ levels.

Individually, and as a collective, the cohort demonstrates that understanding the intersectional SRHR needs of women, men, boys and girls relies on accurate data, use and analysis. Moreover, approaching the data divide as a political, cultural, social and technical challenge requires involving targeted populations through iterative data gathering, analysis and interpretation processes. This data, in turn, is more credible and actionable to address populations’ needs and provides decision-makers with appropriate evidence to inform their policies and programs.

The current polycrisis, with its complex and interlinked crises threatening global health and well-being, necessitates a re-evaluation of global strategies. This is particularly important in the context of the ICPD, which emphasizes the need for comprehensive approaches to reproductive health, gender equality, and sustainable development. The findings from the cohort of research projects in this supplement provide indications of ways forward.

## Data Availability

Not applicable.

## Abbreviations

CSE: Comprehensive Sexuality Education
DALYs: Disability-Adjusted Life Years
HIS: Health Information Systems
hRHR: harmonized Reproductive Health Registry
IDRC: International Development Research Centre
ICPD: International Conference on Population and Development
LMICs: Low- and Middle-Income Countries
SDGs: Sustainable Development Goals
SRH: Sexual and Reproductive Health
WAHO: West African Health Organization
